# Insights from the draft genome of the subsection V (Stigonematales) cyanobacterium *Hapalosiphon* sp. Strain MRB220 associated with 2-MIB production

**DOI:** 10.1186/s40793-016-0175-5

**Published:** 2016-09-02

**Authors:** Boon Fei Tan, Shu Harn Te, Chek Yin Boo, Karina Yew-Hoong Gin, Janelle Renee Thompson

**Affiliations:** 1 Centre for Environmental Sensing and Modelling, Singapore-MIT Alliance for Research and Technology Centre, Singapore, Singapore; 2 NUS Environmental Research Institute, National University of Singapore, Singapore, Singapore; 3 Department of Civil and Environmental Engineering, Massachusetts Institute of Technology, Cambridge, MA USA

**Keywords:** Subsection V cyanobacterium, Stigonematales, *Hapalosiphon*, *Fischerella*, Filamentous heterocysts, 2-MIB, Geosmin synthase

## Abstract

A non-axenic unialgal culture containing a Subsection V (Stigonematales) cyanobacterium, *Hapalosiphon* strain MRB 220, was obtained from a benthic freshwater algal mat through multiple transfers following growth in sterile media. Physiological characterization demonstrated the culture was capable of nitrogen-fixation and production of the off flavor compound 2-methylisoborneol (2-MIB). Total DNA isolated from this culture was sequenced using Illumina HiSeq and *de novo* assembled into contigs. The genome of MRB 220 was separated from co-occurring heterotrophic bacteria using sequence homology and compositional approaches, and its purity was confirmed based on best BLAST hit classification and principle component analysis of the tetranucleotide frequencies of fragmented contigs. The genome of ~7.4 Mbp contains 6,345 protein coding genes with 4,320 of these having functional prediction including predicted pathways for biosynthesis of the secondary metabolite welwitindolinone. Analyses of 16S rRNA gene and whole genome sequence average nucleotide identity indicated close relatedness of MRB 220 to the genera *Hapalosiphon* and *Fischerella* within the order Stigonematales. Microscopic examination showed that MRB 220 formed heterocystous branched filaments, thereby supporting identification of strain MRB 220 as a morphospecies of *Hapalosiphon*. Availability of the draft genome of *Hapalosiphon* strain MRB 220 enables future work to elucidate the pathway and dynamics for biosynthesis of 2-MIB and other secondary metabolites and understand the ecology and physiology of Stigonematales cyanobacteria in tropical freshwaters.

**Electronic supplementary material:**

The online version of this article (doi:10.1186/s40793-016-0175-5) contains supplementary material, which is available to authorized users.

## Introduction

Cyanobacterial blooms occur in many freshwater environments and are among the major causes of diminishing water quality, due in part to the production of secondary metabolites [1, 2]. Some of these secondary metabolites are hepatotoxins which can be life threatening if ingested [3], while others such as 2-methylisoborneol (2-MIB) and geosmin are off flavor compounds that reduce water palatability [4]. Located close to the equator, Singapore has high daily temperature throughout the year, promoting persistent cyanobacterial blooms in eutrophic water bodies [1]. Several events of elevated off flavor concentrations have been recorded in local freshwaters â prompting a need to identify potential cyanobacterial producers in the local aquatic system for a better water treatment and management strategy [5, 6].

Whole genome sequencing (WGS) is emerging as a powerful tool to help identify and resolve the functional characteristics of cyanobacterial species [7–10]. Classification of cyanobacterial taxa has been traditionally done by comparison to morphological features of preserved type specimens and more recently by molecular taxonomy based on nucleotide sequence homology of marker genes (usually 16S or 16S–23S intergenic spacer regions) [11]. Morphological classification based on characteristics of an individual cell, trichome or colony has subdivided the cyanobacterial phylum into five subsections: (I) unicellular, (II) baeocystous, (III) filamentous, (IV) heterocystous, and (V) ramified, where Subsections III to V are taxa forming filaments or trichomes [12]. In contrast to Subsection III cyanobacteria which have no cellular differentiation (consist of only vegetative cells), members of Subsections IV and V are comprised of heterocystous species with some of them forming akinetes occasionally [12]. Members of Subsections IV and V differ in cell division and branching type. Subsection IV representatives divide solely in one plane and form either false-branching or no branching; while Subsection V are true-branched cyanobacteria (analogue to order Stigonematale) capable of dividing in more than one plane and forming hormogones [12]. Representatives of Subsection V include the genera *Hapalosiphon*, *Fischerella*, *Westiella* and *Westiellopsis*. Further morphological characteristics based on branching patterns (T or Y-branching), position of heterocysts and cell arrangement in trichomes (uniseriate vs multiseriate) are used to classify morphospecies to different genera within the subsection [13].

Molecular analysis of the 16SrRNA gene sequence has supported the grouping of Subsection V (Stigonematales) as a monophyletic cluster. However, the same analysis revealed that named genera within the Subsection V cyanobacteria were polyphyletic [14], reflecting a common observation that cyanobacterial groups defined by morphological characteristics may not be phylogenetically coherent [11]. Comparison of genome sequences has great potential to shed additional light on the differentiation of Subsection V cyanobacteria into different taxa. Average nucleotide identities (ANI) of 95–96 % have been suggested as a threshold for recognizing bacterial species [15] and when available provide an additional means to compare organisms to determine whether they may share a species-level designation.

In this study, we have obtained a non-axenic unialgal culture from a tropical water body experiencing algal blooms and concurrent taste and odour problems. The culture produces the off flavor compound 2-MIB and was dominated by a Subsection V cyanobacterium, morphologically identified as a member of the genus *Hapalosiphon*. Using WGS bioinformatic analysis, the genome of the dominant cyanobacterium was extracted from the metagenome and analyzed for genes and gene clusters predicted to encode for cyanobacterial secondary metabolites. As Subsetion V cyanobacterial strains are noted for nitrogen-fixation via specialized heterocyst cells [13, 14], we determined how growth rate, pigment production, and off flavor production varied in response to nitrogen limitation. Comparison of the genome of MRB 220 to those of the most-closely related genome sequenced Subsection V cyanobacteria indicates a high level of DNA relatedness, suggesting that despite different assigned genus names (based on morphological features) these may represent morphotypes of a single species.

## Organism information

### Classification and features

The unialgal culture sequenced in this study contained heterocystous filaments (strain MRB220;) as the sole cyanobacterial species. Strain MRB 220 was isolated from a benthic cyanobacterial mat gathered from a sediment sample of an urban freshwater water body in Singapore. The water body was initially constructed by damming a river mouth and allowing the flushing out of saline water via rainfall. The physicochemical variables at the sampling site at the time of sample collection were: average water temperature 28.6 °C, monthly rainfall 3.31 mm, chlorophyll-a 86.7 μg/L, pH unit 9.1, conductivity 635 uS/cm, turbidity 7.5 NTU, total nitrogen 0.73 mg/L, nitrate 0.06 mg/L, total phosphorus 0.038 mg/L, dissolved phosphorus 0.0015 mg/L and ammonia 0.04 mg/L. A sediment corer was used to collect a benthic sample at a water depth of 4 m, after which the top 2 cm of the sediment layer was scraped and suspended in sterile MLA medium [16] for microscopic observation. Subsequently, individual cyanobacterial trichomes representing Stigonematales filaments were aseptically picked and transferred to sterile water droplets using a micropipette. The washing step was repeated until a single trichome free from other algal cells/filaments was obtained. Individual trichomes were subsequently transferred to MLA medium [16] containing sodium nitrate and dipotasium phosphate used as the main sources of nitrogen and phosphorus for growth. Cultures were incubated at 25 °C with 12:12 day/night cycle at a light intensity of 20 ± 5 μmol quanta mˉ^2^sˉ^1^. After two successive transfers in MLA medium with sodium nitrate, the culture was grown in modified MLA medium [16] without nitrogen source to selectively enrich for nitrogen-fixing cyanobacteria, e.g., *Hapalosiphon* against other non-nitrogen-fixing algal species.

**Fig. 1:**
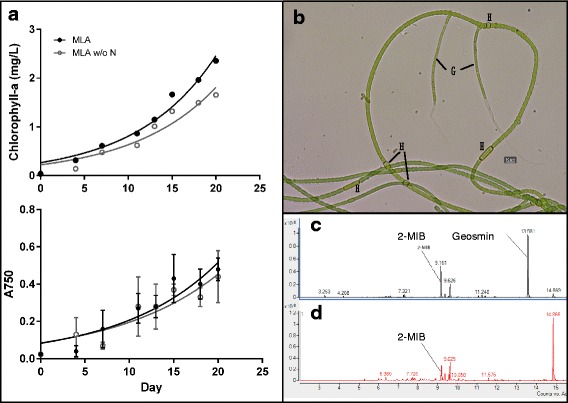
**a** Growth curve of strain MRB 220 grown in MLA medium with and without (w/o) nitrogen source, measured by the content of chlorophyll-a (*Top*), and optical density (OD, *bottom*); **b** Bright field micrograph of strain MRB 220 – H: heterocyst, G: hormogonium; **c** HS-SPME GC-MS/MS chromatograms for geosmin and 2-MIB standards and liquid culture of MRB 220 (**d**)

The growth rate of subcultures grown with or without nitrogen source was inferred based on chlorophyll-a and optical density (OD) measurements (Fig. 1a). Nitrogen availability had insignificant effect on the growth of the culture, indicating that strain MRB 220 was capable of nitrogen fixation, consistent with previous findings that showed members of the Stigonematales are nitrogen fixers [17]. The response of MRB 220 under nitrogen starvation was compared by examining differences in cell size and pigment content. Cells grown in N-enriched and N-free media for 2 months were harvested and visualized under an inverted microscope Leica DFC450 C). Photosynthetic pigments, i.e. chlorophyll-a (chl-a) and phycobiliproteins (phycocyanin, PC; allophycocyanin APC and phycoerythrin, PE) were extracted, measured with a spectrophotometer [18] and normalized to freeze-dried cell weight (DW). The vegetative cells of MRB 220 were in barrel or cylindrical shape (length: 6.8 ± 1.5 μm; width: 3.5 ± 0.5 μm) while the heterocysts were spherical, barrel or cylindrical in shape (length: 6.8 ± 1.5 μm; width: 3.5 ± 0.5 μm). Heterocysts were present in both N-free and -enriched media, but no akinete was observed. The vegetative cell : heterocyst ratios were approximately 50:1 in N-free medium and 120:1 in N-enriched medium. In addition, nitrogen availability was found to have insignificant effect on the vegetative and heterocyst cell sizes. Under a N-enriched environment, pigment contents of the culture were 12.30, 2.08, 2.71 and 12.37 mg/g DW for chl-a, PC, APC and PE, respectively, making up ~3 % of the cell DW. Notably, a 2-fold increase of all phycobiliproteins was observed for the culture in N-free medium (Independent T-test, *p* < 0.01), consistent with other studies on cyanobacterial pigment analyses [19]. Phycobiliproteins are accessory pigments harboured by cyanobacteria and responsible for the light harvesting mechanism in photosystem II [20]. They are also reserved in the cell, providing a nitrogen source when nitrogen becomes deficient in the environment [21], which explains the accumulation of phycobiliproteins of MRB 220 under nitrogen starvation observed in our study.

Heterocysts of MRB220 were distinguished from vegetative cells and akinites (dormant cells) by their larger cell size, thicker cell wall and less granular cytoplasm under bright-field or phase-contrast microscopy. Akinites were observed as larger cells with high granular content, appearing darker by microscopic observation (Fig. 1b). In addition, co-occurrence of heterotrophic bacteria was observed by microscopy and repeated attempts to further purify the algal strain from these bacteria by dilution and serial transfer were unsuccessful. Many cyanobacterial cultures that have been previously characterized by bioassays and whole genome sequencing have been co-cultures of a single algal strain with companion heterotrophic bacterial communities, suggesting strong association of some cyanobacterial species with heterotrophic bacteria [10, 22, 23].

Many filamentous cyanobacteria are known to produce a vast variety of secondary metabolites, some of which are odorous compounds that can diminish the aesthetic quality of potable or recreational waters. To determine potential off flavor production of MRB 220, the strain was grown to early stationary phase and the culture fluid was collected and analyzed using a HS-SPME GC-MS/MS method developed previously [24]. Using this method, we analysed the presence of two off flavor compounds in the culture fluids, i.e. 2-MIB and geosmin. A total of eight calibration standards ranging from 0 to 500 ng/L were prepared from the combined standard solution of 2-MIB, geosmin, β-cyclocitral and β-ionone. The detection limit for each of the four compounds was 5 ng/L. Ten milliliters of the calibration standards and MRB 220 culture fluid were added in separate GC vials containing 1 % v/v of sodium chloride and subsequently spiked with 100 ppt of β-cyclocitral-d5 for internal calibration purposes. The samples were analyzed without delay using an Agilent GC 7890A coupled with 7000B Triple Quad series mass spectral detector. Only 2-MIB was detected in both cultures cultivated in N-enriched and N-free media at concentrations of 120 ng Lˉ^1^ and 80 ng Lˉ^1^, respectively (Fig. 1c). Our result indicated that MRB 220 was able to synthesize 2-MIB even under nitrogen limiting conditions, but was not able to synthesize geosmin in either nitrogen free or nitrogen depleted media.

**Fig. 2:**
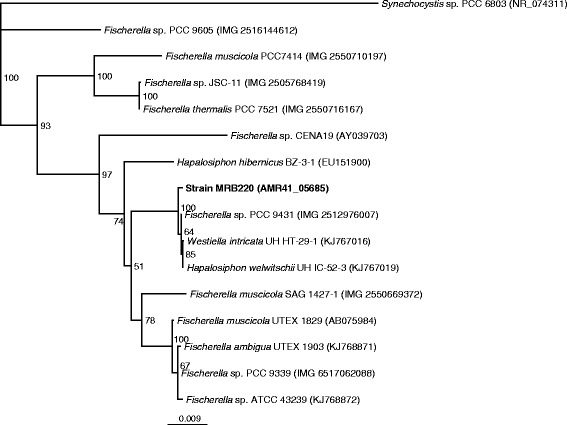
Neighbor-joining tree of the 16S rRNA gene of MRB220 and selected 16S rRNA gene sequences from members of Stigonematales. All 16S rRNA sequences were aligned with MUSCLE [49], and neighbor-joining analysis with 100 bootstrap replicates was conducted using Geneious Tree-builder [50] based on the Tamura-Nei Model. Bootstrap values are labelled in each branch note and *Synechocystis sp.* PCC 6803 was used as the outgroup

The single copy 16S rRNA gene of MRB 220 is 1,414 bp and has > 99 % identity to those in *Hapalosiphon welwitschii* UH IC-52-3 (KJ767019.1), *Westiella intricate* UH HT-29-1 (KJ767016) and *Fischerella* sp. PCC 9431 (FIS9431_RS0103235) which together form a phylogenetic clade among the Subsection V cyanobacteria (Fig. 2); supported by the pair-wise average nucleotide identity of > 97 % identity of the whole genome sequences (Additional file 1: Table S1). The phylogenetic relatedness of MRB 220, UH IC-52-3, UH HT-29-1 and PCC 9431 was also confirmed by analysis of the 16S–23S rRNA internally transcribed spacer region (Additional file 2: Figure S1). Phylogenetic clustering and high sequence similarity of the 16S rRNA gene and the ITS region of MRB 220 with strains assigned to the genera *Hapalosiphon*, *Westiella* and *Fischerella* precluded identification to the genus level. The genera *Hapalosiphon*, *Westiella* and *Fischerella* each possess unique morphological characteristics that enable taxonomic classification [13] Unique features of Strain MRB 220 were examined microscopically. Morphological traits, such as having creeping and erect thallus, T-branching, cells in the main axis were uniseriate, and main axis and lateral branches with similar cell size and shape (Fig. 1b), together suggest that MRB220 belongs to the genus of *Hapalosiphon* (Table 1) [14, 25].

**Table 1:** Classification and general features of Strain MRB 220 according to MIGS recommendations [51]

MIGS ID	Property	Term		Evidence code^a^
	Current classification	Domain	*Bacteria*	TAS [52]
		Phylum	*Cyanobacteria*	TAS [12]
		Class	*Cyanophyceae*	TAS [14, 25]
		Subsection	V	
		Genus	*Hapalosiphon*	
		Species	Unknown	
		Type	Strain MRB 220	
	Gram stain	Gram negative		TAS [53]
	Cell shape	Filamentous/thallous		IDA
	Motility	Attached to substrate or free-floating		IDA
	Sporulation	Not reported		
	Temperature range	Not reported		
	Optimum temperature	Not reported		
	pH range; Optimum	Not reported		
	Carbon source	Not reported		
	Energy source	Phototrophic		IDA
	Terminal electron receptor	Not reported		
MIGS-6	Habitat	Freshwater		IDA
MIGS-6.3	Salinity	0.33 ppt		
MIGS-22	Oxygen requirement	Aerobic		IDA
MIGS-15	Biotic relationship	Free living		IDA
MIGS-14	Pathogenicity	Non-pathogen		IDA
MIGS-4	Geographic location	Southern region, Singapore		IDA
MIGS-5	Sample collection time	July, 2013		IDA
MIGS-4.1	Latitude	1.287718		IDA
MIGS-4.2	Longitude	103.866195		IDA
MIGS-4.3	Depth	3 m		IDA
MIGS-4.4	Altitude	Not applicable		

^a^Evidence codes - IDA: Inferred from Direct Assay; TAS: Traceable Author Statement (i.e., a direct report exists in the literature); NAS: Non-traceable Author Statement (i.e., not directly observed for the living, isolated sample, but based on a generally accepted property for the species, or anecdotal evidence). These evidence codes are from the Gene Ontology project [54]

## Genome sequencing information

### Genome project history

The project information and associated MIGS 2.0 compliance [26] are provided in Table 2. This organism was selected for sequencing as it was determined to be one of the most commonly found benthic cyanobacteria that contribute to algal blooms in tropical freshwater environments in Singapore and was associated with production of the off flavor compound 2-MIB in laboratory studies. This work provided a standard draft genome and the assembled contigs have been deposited in the NCBI database and Joint Genome Institute under the accession LIRN00000000 and Ga0082282, respectively.

**Table 2:** Genome sequencing project information

MIGS ID	Property	Term
MIGS-31	Finishing quality	Draft
MIGS-28	Libraries used	Illumina Truseq Nano DNA Library Prep Kit
MIGS-29	Sequencing platforms	HiSeq Rapid V2 sequencing Run
MIGS-31.2	Fold coverage	150X
MIGS-30	Assemblers	CLC Genomics Workbench 8.0
MIGS-32	Gene calling method	Prodigal
	Locus Tag	AMR41
	Genbank ID	LIRN00000000
	Genbank Data of Release	August 29, 2015
	Gold ID	Ga0082282
MIGS-13	Bioproject	PRJNA224116
	Source material identifier	MRB220
	Project relevance	Cyanobacterial ecology, Environmental

### Growth conditions, and genomic DNA preparation

The non-axenic unialgal culture containing strain MRB 220 was grown in MLA medium without nitrogen source and incubated for growth as discussed above at 25 °C and light intensity of 20 ± 5 μmol quanta mˉ^2^sˉ^1^. Upon reaching stationary phase, total DNA was isolated from this culture using a PowerSoil DNA Isolation Kit (Mo Bio) and the DNA concentration was quantified using a Qubic fluorometer (Life Technologies, USA) following the manufacturer’s instructions.

### Genome sequencing and assembly

**Fig. 3:**
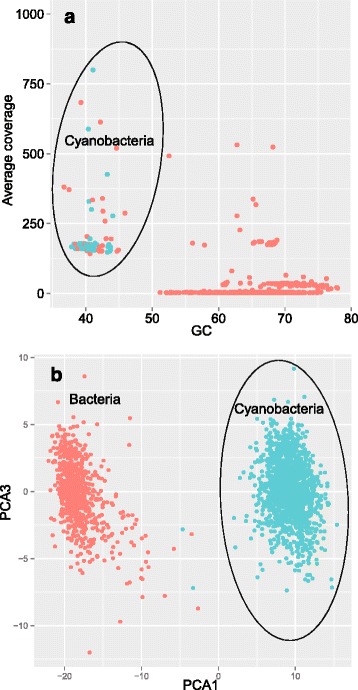
**a** Contig coverage versus GC content. Each *dot* represents a single contig of >1000 bp. Contig coverage was estimated using CLC Genomics Workbench by mapping assembled contigs with Illumina reads that shared 96 % similarity over 95 % sequence *red* length. *Green color dots* represent contig having single copy gene/s that have taxonomic affiliation with cyanobacteria. **b** Principle component analysis of the tetranucleotide frequencies of assembled contigs fragmented to 1,000 bp. *Green color dots* represent contigs that have affiliation with cyanobacteria, binned using method in (**a**). All binned contigs have at least 50 % of their open reading frames annotated as being affiliated with cyanobacteria based on best BLAST hits

The total DNA was used in the construction of a paired-end library constructed with Illumina Truseq Nano DNA Library Prep Kit, and sequenced using Illumina HiSeq 2000 applying the 250 bp paired-end sequencing protocol at Singapore Centre for Environmental Life Sciences Engineering. Quality control of Illumina paired-end raw reads was conducted using CLC Genomics Workbench V.8 (CLC-Bio, USA) by removing adaptors and reads with quality score <0.01 and length <150 bp. Following this, paired-reads were subjected to *de novo* assembly with CLC Genomics Workbench V8.0 using default kmer size. The mini-metagenome was assembled into 1,512 contigs (1,008– 810,851 bp) with N_50_ of 137,929 bp and average length of 11,246 bp. The genome of strain MRB 220 was extracted from the mini-metagenome using both sequence compositional and homology-based approaches. First, all open reading frames of the assembled metagenome were predicted using MetaProdigal [27], after which single copy genes were identified using Hidden Markov Models [28] followed by BLASTX searches implemented in Diamond [29] and taxonomic assignment using MEGAN 5 [30]. Overlaying taxonomic affiliation of single copy genes on a “contig coverage versus GC plot” showed that only a single cyanobacterium with GC content of 35–45 % and contig coverage >100 was present in the metagenome. The co-occurring heterotrophic bacteria consist of a dominant *Xanthomonadaceae* and other minor heterotrophic bacteria (GC contents of 50–80 %; Fig. 3a). Contigs putatively assigned to the cyanobacterial genomic bin were extracted from the metagenome and verified for the presence of non-cyanobacteria contigs using a BLASTX-based approach. Briefly, all contigs were *in silico* fragmented to 1000 bp, followed by BLASTX searches against the NCBI NR-database using Diamond and taxonomic assignment using MEGAN, by which no sequence contamination was identified. The purity of the genome representing the cyanobacterium was also confirmed using approaches in tetranucleotide frequencies followed by principle component analyses (Fig. 3b). The detected number of single copy genes indicated that the draft genome of MRB 220 was nearly complete (100 %).

### Genome annotation

Gene predication was performed using Prodigal [31] as part of the Joint Genome Institute automated genome annotation pipeline [32] and the NCBI Prokaryotic Genome Annotation Pipeline [33]. Additionally, gene clusters encoding secondary metabolite biosynthesis were predicted using AntiSMASH 3.0 [34].

## Genome properties

**Fig. 4:**
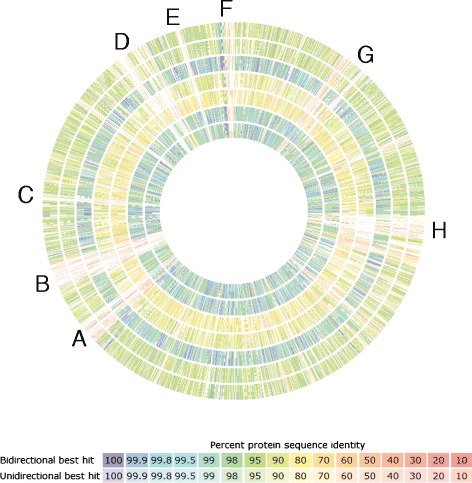
Genomics comparison of MRB 220 to other Stigonematales genomes conducted using RAST [35]. Each track represents pair-wise BLAST similarity between the open reading frames in query genome against those in MRB 220, with percentage of similarity represented with different colors shown in the legend. Regions marked in the genomic map correspond to gene number presented in Additional file 1: Table S1 (*A* = 4521–4555; *B* = 4806–4972, *C* = 5343–5358, *D* = 6290–6493, *E* = 6688–6724, *F* = 6974–7032, *G* = 665–879, *H* = 1788–1993). Query genomes used in this analyses, from outer ring: *Fischerella muscicola* PCC 7414, *Fischerella muscicola* SAG 1427-1, *Fischerella* PCC 9431, *Fischerella* sp. JSC-11*, Fischerella* sp. PCC 9605, *Hapalosiphon welwitschii* and *Westiella intricate UH HT-29-1*

The draft genome of MRB 220 is 7.4 Mbp with 40.2 % GC content, similar to genomes of other Stigonematales [7, 9], and contained in 115 scaffolds (1,055–810,851 bp) with N_50_ of 108,475 bp (Table 3). Annotation using the DOE IMG pipeline [32] predicted 6,345 coding sequences (Table 3) with 4,320 having functional prediction, whereas annotation using the RAST pipeline [35] predicted 7032 protein coding sequences with 2831 being annotated as hypothetical proteins. COG annotation of protein coding genes is presented in Table 4. Overview of the genome of MRB 220 in comparison to other sequenced Stigonematales genomes is presented in Fig. 4.

**Table 3:** Genome statistics for Strain MRB 220

Attribute	Value	Percent of total
Genome size (bp)	7,429,720	100.0
DNA coding region (bp)	6,007,607	81.0
DNA G + C	2,985,683	40.2
DNA scaffolds	115	-
Total genes	6,404	100.0
Protein-coding genes	6,345	99.1
RNA genes	59	0.9
Pseudo genes	304	4.7
rRNA operons^a^	4	0.1
Genes with function prediction	4,320	67.5
Genes assigned to COGs	3,342	52.2
Genes assigned pfam domains	4,652	72.6
Genes with signal peptides	201	3.1
Genes with transmembrane proteins	1,572	24.6
CRISPR repeats	7	-

^a^1 copy of 5S, 1 copy of 16S and 2 copies of 23S rRNA (data obtained using the JGI IMG pipeline)

**Table 4:** Number of genes associated with general COG functional categories

Code	Value	Percent of total	Description
J	206	5.6	Translation, ribosomal structure and biogenesis
A	-	-	RNA processing and modification
K	155	4.2	Transcription
L	115	3.1	Replication, recombination and repair
B	2	0.1	Chromatin structure and dynamics
D	47	1.3	Cell cycle control, cell division, chromosome partitioning
V	138	3.7	Defense mechanisms
T	266	7.2	Signal transduction mechanisms
M	292	7.9	Cell wall/membrane biogenesis
N	57	1.5	Cell motility
W	19	0.5	Extracellular structures
U	41	1.1	Intracellular trafficking and secretion
O	171	4.6	Posttranslational modification, protein turnover, chaperones
C	201	5.5	Energy production and conversion
G	195	5.3	Carbohydrate transport and metabolism
E	239	6.5	Amino acid transport metabolism
F	72	2.0	Nucleotide transport and metabolism
H	235	6.4	Coenzyme transport and metabolism
I	112	3.0	Lipid transport and metabolism
P	247	6.7	Inorganic ion transport and metabolism
Q	117	3.2	Secondary metabolite biosynthesis, transport and catabolism
R	479	13.0	General function prediction only
X	57	1.5	Mobilome: prophages, transposons
S	227	6.2	Function unknown
-	3062	47.8	Not in COGs

## Insights from the genome sequence

The genome of MRB 220 is highly similar to those of *Hapalosiphon welwitschii* UH IC-52-3, *Fischerella*PCC 9431 and *Westiella intricate* UH HT-29-1 based on average nucleotide identity (>97 %, Additional file 1: Table S1), shared gene content (Additional file 3: Table S2) and supported by phylogenetic analyses of the 16S rRNA gene and 16S–23S rRNA ITS (Fig. 2 and Additional file 2: Figure S1). The average nucleotide similarity shared among these four strains is above the threshold recommended for designation to the same bacterial species [15] and is consistent with the inability of phylogenetic analysis of the 16S rRNA gene or ITS region to resolve the genus groupings of these strains. Comparison between the genomes of MRB 220 and the three most closely related Stigonematales by bi- or uni- directional best BlastP implemented in RAST and cross validated with IMG annotations revealed strain-specific genes that primarily encode hypothetical proteins (Fig. 4, Additional file 3: Table S2). The strain-specific ORFs with annotated gene functions in MRB220 include a high proportion of predicted phage-like proteins, mobile genetic elements, and membrane associated proteins pointing to the significance of evolutionary selective pressures at the cyanobacterial cell wall. Additional strain specific genes are predicted to mediate DNA replication, regulation of gene expression and sugar metabolism. Finally, a strain-specific toxin-antitoxin system (RelBE), a siderophore permease, and a complete Hox hydrogenase operon (HoxEFUYH) (related strains contain only the HoxH gene) may reflect strain-specific adaptation for cell differentiation/apoptosis [36], iron-uptake, and hydrogen metabolism [37], respectively (Additional file 3: Tables S2 and S3).

**Fig. 5:**
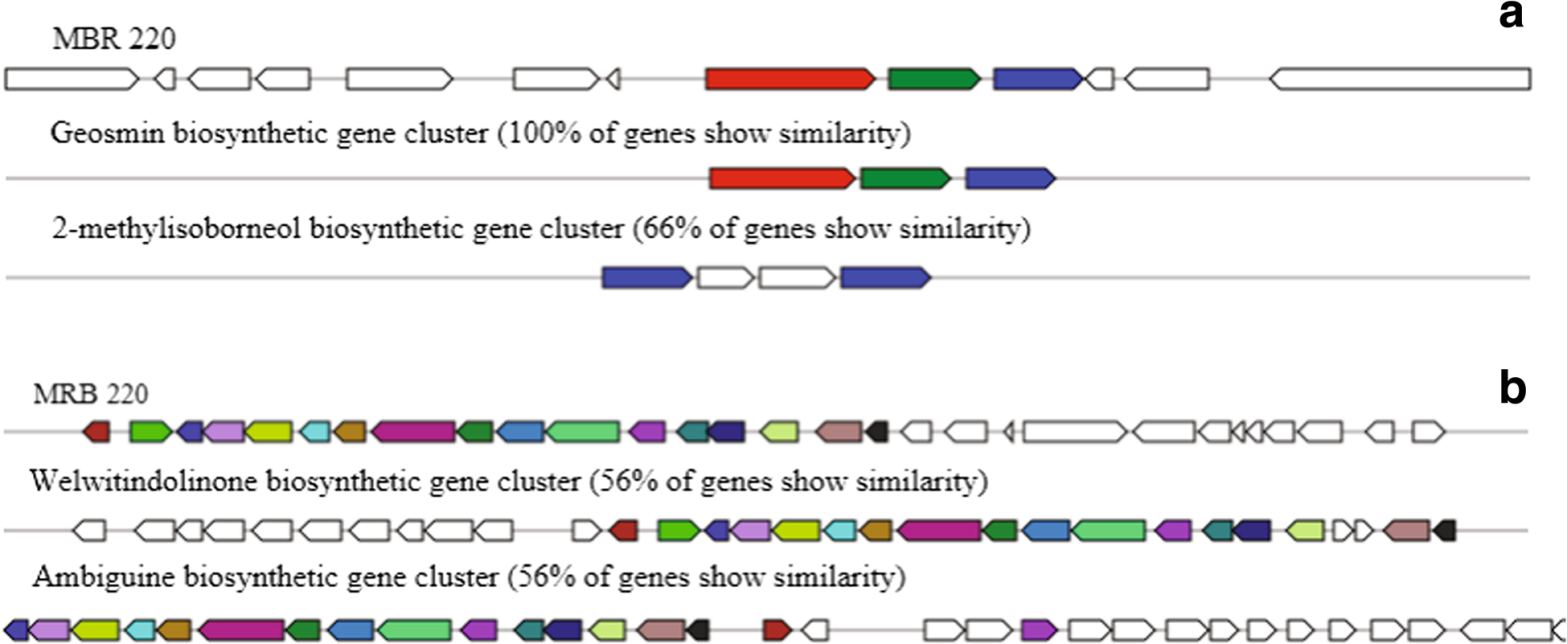
Gene cluster in the genome of MRB 220 encoding biosynthesis of **a** geosmin, and **b** welwitindolinone, detected using antiSMASH 3.0 [34]. Geosmin synthase detected in the gene cluster was disrupted with insertional sequences (Additional file 4: Figure S2), and two genes (*blue*) share sequence similarity to those present in gene cluster encoding 2-methylisoborneol

Members of the Stigonematales are known to produce secondary metabolites including hapalosin, bacteriocin, terpene, and welwitindolinone that have biotechnological applications [8, 9, 38]. To date, off flavors synthesis ability of *Hapalosiphon* spp. remains unknown, although related strains such as *Fischerella**muscicola* (of which the genome has not been sequenced) are known to produce geosmin [39]. Genome mining for secondary metabolite biosynthesis gene clusters in MRB 220 using antiSMASH 3.0 [34] identified complete and partial gene clusters predicted to encode biosynthesis of geosmin, puwainaphycin, hectochlorin and welwitindolinone. While certain strains of *Hapalosipon* sp. [40] and *Fischerella* sp. [41, 42] are capable of producing the toxin microcystin, microcystin biosynthesis genes were not detected in MRB 220 (by PCR and genome analysis) nor in other Stigonematales genomes previously reported [8, 9]. A partial gene cluster (56 % of genes; ~17 of 31 genes) encoding welwitindolinone was detected in a single contig in the genome of MRB 220 and it was highly conserved compared to the gene clusters detected in *Fischerella* sp. and *Hapalosiphon* sp. [8] (Fig. 5) which were distinguished by gene additions. Although no geosmin was detected in cultures, a complete gene cluster predicted to encode biosynthesis of the off flavor geosmin was observed in MRB 220 and contains a terpene synthase (Locus_tag: AMR41_20665) and two genes encoding cyclic nucleotide-binding domain located downstream (AMR41_20670, AMR41_20675; Fig. 5).

Similar geosmin biosynthesis gene clusters are found in the genomes of closely related cyanobacteria and are associated with variable observed activity of geosmin synthesis. Genomes of *W. intricata* UH strain HT-29-1, *H. welwitschii* UH strain IC-52-3, *Fischerella* sp. PCC 9431 and *F. muscicola*SAG 1427–1 [9] contain predicted geosmin biosynthesis gene clusters, with key enzyme geosmin synthase sharing high amino acid homology (>90 %) to those in geosmin-producers “*Nostoc punctiforme*” PCC 73102 [43] and *Calothrix*PCC 7507 [44]. However, through several experiments these strains have not demonstrated geosmin production [8, 9]. The geosmin synthase homolog AMR41_20665 detected in MRB 220 (by antiSMASH and RAST) shares low sequence similarity (<49 %) to those detected in geosmin-producing strains and moreover lacks two strictly conserved motifs for Mg2+ binding that are found in all sesquiterpene and monoterpen synthases [45] (Additional file 4: Figure S2) suggesting that the enzyme produced by the annotated gene is inactive. Indeed, inactivation of secondary metabolite gene clusters in certain cyanobacterial species is common and can occur through deletional, insertional and point mutations within gene clusters [46, 47].

Metabolite analysis detected the presence of 2-MIB in MRB 220 culture fluids (Fig. 1c). A complete 2-MIB biosynthesis gene cluster consists of three genes (i.e. genes encoding SAM-dependent methyltransferase, monoterpene cyclase or terpene synthase, and nucleotide-binding protein) [48]. The genome of MRB 220, and the metagenome of the companion bacterial flora, was screened for genes from the 2-MIB gene cluster using antiSMASH [34], the RAST annotation pipeline [35] and by comparison to reference genes of the 2-MIB cluster from *Planktothricoides raciborskii*CHAB3331. Only one out of the three genes associated with the 2-MIB biosynthesis cluster was observed in the draft genome of MRB 220, represented by two coding sequences annotated as cAMP-binding proteins and located in the predicted geosmin biosynthesis gene cluster (AMR41_20670 and AMR41_20675) sharing ~62 % amino acid similarity to the nucleotide-binding proteins from *Planktothricoides raciborskii*CHAB3331 (HQ830029). The presence of sequence homologs involved in the geosmin and 2-MIB production is unsurprising as they are known to share certain common biosynthesis pathways [38]. Additional genes annotated by RAST as terpene synthase (AMR41_18580) and squalene cyclase (AMR41_02940) did not have significant homology to known 2-MIB pathway genes. Determining which genes are involved in the biosynthesis of 2-MIB in strain MRB 220 is a subject of future investigation.

## Conclusions

Genomic and physiological characterization identify *Hapalosipon* strain MRB220 as a heterocyst-forming filementous cyanobacteria capable of nitrogen-fixation and associated with production of the off flavor compound 2-MIB under nitrogen-free and replete conditions. High average nucleotide identities between genomes from *Hapalosiphon* strain MRB220 and individual strains from the genera *Hapalosiphon*, *Westiella* and *Fischerella* are consistent with a shared species-level designation and suggest that further work is needed to reconcile genus and species assignment based on morphological characteristics with emerging molecular and genomic data. In addition, availability of the draft genome of strain MRB 220 will help future work to understand the pathway and dynamics for biosynthesis of 2-MIB and other secondary metabolites as well as the ecology and physiology of this strain in tropical freshwaters.

## Additional files

Additional file 1: Table S1. Average nucleotide identity (ANI) between genome of MRB 220 and those of other Stigonematales. Note: Average nucleotide identity was computed using the JGI IMG pipeline [32]. (DOC 38 kb) Additional file 2: Figure S1. Neighbor-joining tree of the 16S–23S rRNA internal transcribed spacer of MRB220 and selected gene sequences extracted from sequence genomes of Stigonematales. All sequences were aligned with MUSCLE [49], and alignment column <70 % identity was stripped using Geneious R7 [50]. Neighbor-joining analysis with 100 bootstrap replicates was conducted using Geneious Tree-builder based on the Tamura-Nei Model. Bootstrap values are labelled in each branch node. (PDF 118 kb) Additional file 3: Table S2. BLAST comparison between Open reading frames of MRB 220 and those in query genomes. **Table S3.** Open reading frames present in the genome of MRB220 but not detected in the three most closely related Stigonematales. (XLSX 2467 kb) Additional file 4: Figure S2. Sequence alignment of geosmin synthase (geoA) a key enzyme in the pathway for geosmin biosynthesis. Nucleotide alignment was conducted using MUSCLE [49] implemented in Geneious R9 [50]. The height of letters in the sequence logo indicated the degree to which the sequence is conserved. While the annotated geoA sequence from MRB 220 reveals partial nucleotide homology, strictly conserved Mg2+ binding sites at position 90 (DDHFLE) and position 476 (DDYFP) are not shared by MRB 220 indicating the enzyme is likely inactive. (PDF 2328 kb)

## Abbreviations

2-MIB: 2-methylisoborneol
APC: Allophycocyanin
chl-a: Chlorophyll-a
DW: Freeze-dried cell weight
GC-MS: Gas chromatography mass spectrophotometry
HS-SPME: Headspace-solid phase microextraction
PC: Phycocyanin
PE: Phycoerythrin
