# Supplementary material for: Insights from the draft genome of the subsection V (Stigonematales) cyanobacterium Hapalosiphon sp. Strain MRB220 associated with 2-MIB production
Source: Stand Genomic Sci. 2016 Sep 2;11(1):58. doi: 10.1186/s40793-016-0175-5 (PMC5009524; doi:10.1186/s40793-016-0175-5)
Supplement: Additional file 1: Table S1. — Average nucleotide identity (ANI) between genome of MRB 220 and those of other Stigonematales. Note: Average nucleotide identity was computed using the JGI IMG pipeline [32]. (DOC 38 kb) [file 40793_2016_175_MOESM1_ESM.doc]

**Table S1.** Average nucleotide identity (ANI) between genome of MRB 220 and those of other Stigonematales.

| **Genome 1** | **Genome 2** | **ANI** |
| --- | --- | --- |
| *Westiella intricata* UH HT-29-1 | *Hapalosiphon* sp. MRB 220 | 97.98 |
| [*Fischerella*](http://doi.org/10.1601/nm.767) sp. [PCC 9431](http://doi.org/10.1601/strainfinder?urlappend=%3Fid%3DPCC+9431) | *Hapalosiphon* sp. MRB 220 | 97.94 |
| *Hapalosiphon welwitschii* UH strain IC-52-3 | *Hapalosiphon* sp. MRB 220 | 97.92 |
| [*Fischerella*](http://doi.org/10.1601/nm.767) *muscicola* [SAG 1427-1](http://doi.org/10.1601/strainfinder?urlappend=%3Fid%3DSAG+1427-1) | *Hapalosiphon* sp. MRB 220 | 92.5 |
| [*Fischerella*](http://doi.org/10.1601/nm.767)sp. [PCC 9339](http://doi.org/10.1601/strainfinder?urlappend=%3Fid%3DPCC+9339) | *Hapalosiphon* sp. MRB 220 | 91.68 |
| [*Fischerella*](http://doi.org/10.1601/nm.767) *muscicola* [PCC 7414](http://doi.org/10.1601/strainfinder?urlappend=%3Fid%3DPCC+7414) | *Hapalosiphon* sp. MRB 220 | 87.24 |
| [*Fischerella*](http://doi.org/10.1601/nm.767) sp. JSC-11 | *Hapalosiphon* sp. MRB 220 | 87.04 |
| “[*Fischerella thermalis*](http://doi.org/10.1601/nm.768)” [PCC 7521](http://doi.org/10.1601/strainfinder?urlappend=%3Fid%3DPCC+7521) | *Hapalosiphon* sp. MRB 220 | 87.02 |
| [*Fischerella*](http://doi.org/10.1601/nm.767) sp. [PCC 9605](http://doi.org/10.1601/strainfinder?urlappend=%3Fid%3DPCC+9605) | *Hapalosiphon* sp. MRB 220 | 81.32 |

Note: Average nucleotide identity was computed using the JGI IMG pipeline [33]
