# Supplementary figures and images for: Insights from the draft genome of the subsection V (Stigonematales) cyanobacterium Hapalosiphon sp. Strain MRB220 associated with 2-MIB production
Source: Stand Genomic Sci. 2016 Sep 2;11(1):58. doi: 10.1186/s40793-016-0175-5 (PMC5009524; doi:10.1186/s40793-016-0175-5)

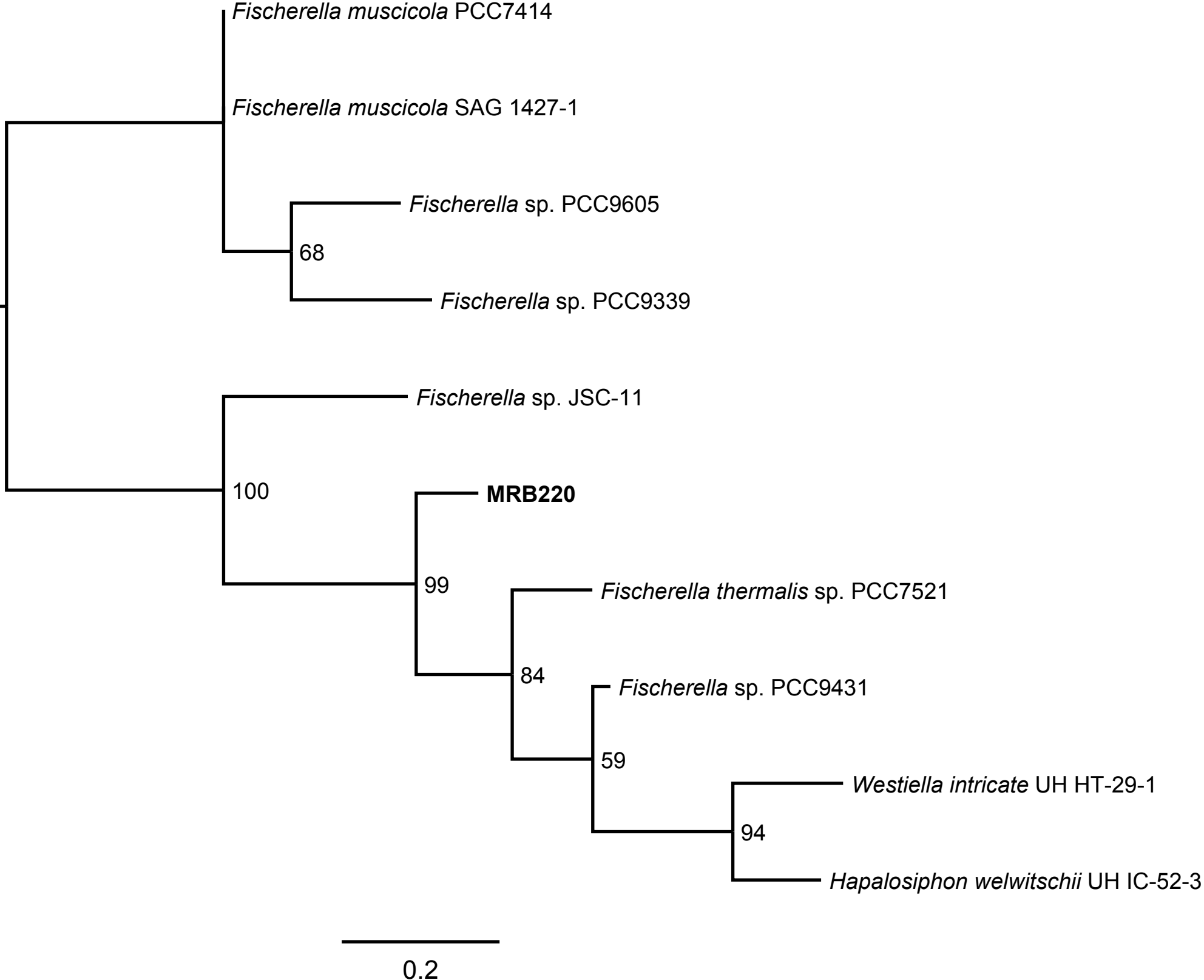

Supplement: Additional file 2: Figure S1. — Neighbor-joining tree of the 16S–23S rRNA internal transcribed spacer of MRB220 and selected gene sequences extracted from sequence genomes of Stigonematales. All sequences were aligned with MUSCLE [49], and alignment column <70 % identity was stripped using Geneious R7 [50]. Neighbor-joining analysis with 100 bootstrap replicates was conducted using Geneious Tree-builder based on the Tamura-Nei Model. Bootstrap values are labelled in each branch node. (PDF 118 kb) [file 40793_2016_175_MOESM2_ESM.pdf]
