# Supplementary material for: Insights from the draft genome of the subsection V (Stigonematales) cyanobacterium Hapalosiphon sp. Strain MRB220 associated with 2-MIB production
Source: Stand Genomic Sci. 2016 Sep 2;11(1):58. doi: 10.1186/s40793-016-0175-5 (PMC5009524; doi:10.1186/s40793-016-0175-5)
Supplement: Additional file 4: Figure S2. — Sequence alignment of geosmin synthase (geoA) a key enzyme in the pathway for geosmin biosynthesis. Nucleotide alignment was conducted using MUSCLE [49] implemented in Geneious R9 [50]. The height of letters in the sequence logo indicated the degree to which the sequence is conserved. While the annotated geoA sequence from MRB 220 reveals partial nucleotide homology, strictly conserved Mg2+ binding sites at position 90 (DDHFLE) and position 476 (DDYFP) are not shared by MRB 220 indicating the enzyme is likely inactive. (PDF 2328 kb) [file 40793_2016_175_MOESM4_ESM.pdf]

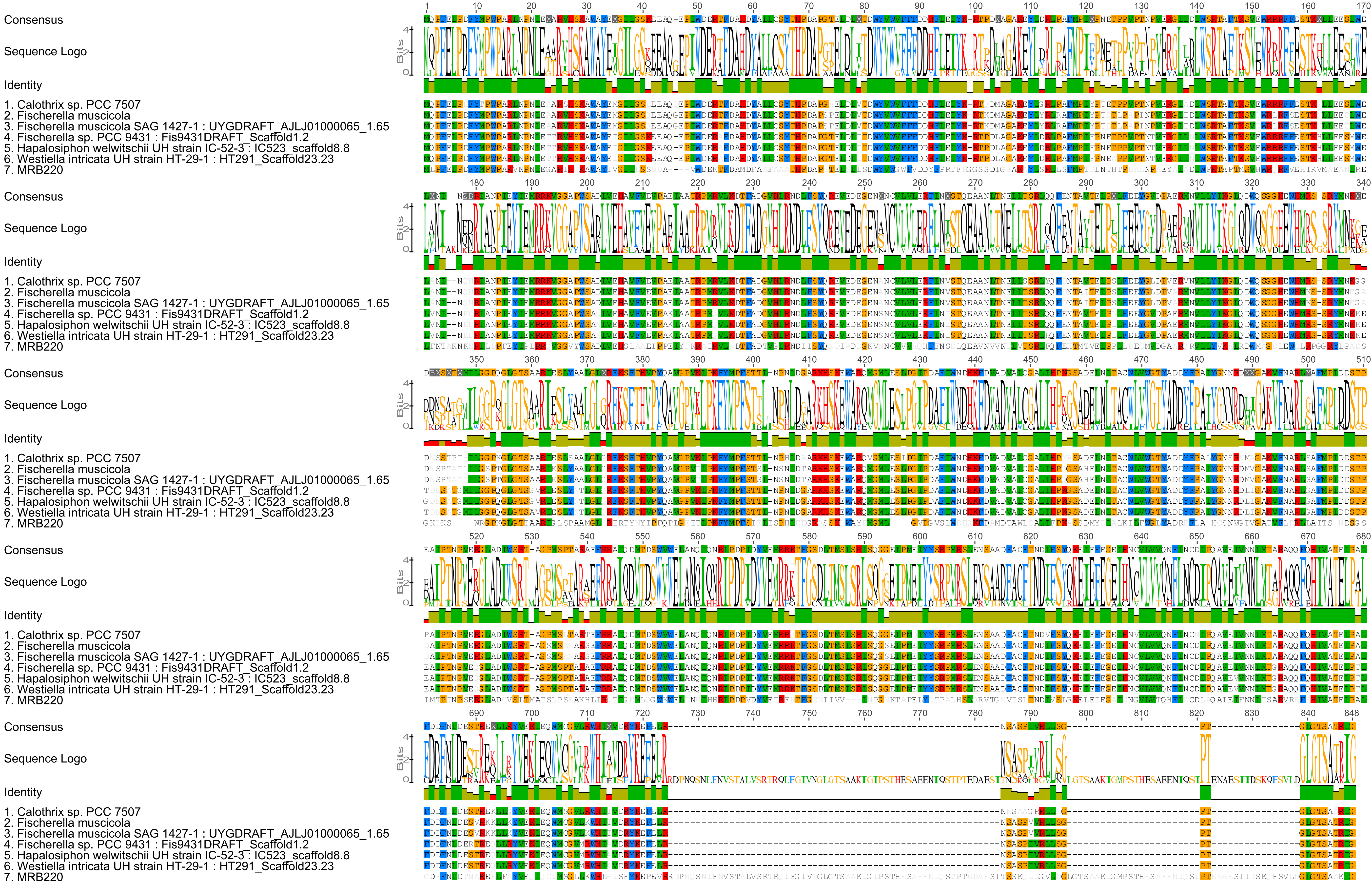

Consensus
Sequence Logo
Identity
1. Calothrix sp. PCC 7507
2. Fischerella muscicola
3. Fischerella muscicola SAG 1427-1 : UYGDRAFT_AJLJ01000065_1.65
4. Fischerella sp. PCC 9431 : Fis9431DRAFT_Scaffold1.2
5. Hapalosiphon welwitschii UH strain IC-52-3 : IC523_scaffold8.8
6. Westiella intricata UH strain HT-29-1 : HT291_Scaffold23.23
7. MRB220
Bits
